# Fatigue In Teenagers on the interNET - The FITNET Trial. A randomized clinical trial of web-based cognitive behavioural therapy for adolescents with chronic fatigue syndrome: study protocol. [ISRCTN59878666]

**DOI:** 10.1186/1471-2377-11-23

**Published:** 2011-02-19

**Authors:** Sanne L Nijhof, Gijs Bleijenberg, Cuno SPM Uiterwaal, Jan LL Kimpen, Elise M van de Putte

**Affiliations:** 1 Department of Pediatrics, Wilhelmina Children's Hospital, University Medical Centre Utrecht, The Netherlands; 2 Expert Centre for Chronic Fatigue, Radboud University Nijmegen Medical Centre, The Netherlands; 3 Julius Centre for Health Sciences and Primary Care, University Medical Centre Utrecht, The Netherlands

## Abstract

**Background:**

Chronic Fatigue Syndrome (CFS) is increasingly recognized as a cause of disability and inactivity in adolescents in the Netherlands. CFS is characterized by unexplained fatigue lasting more than 6 months. Cognitive Behavioural Therapy (CBT) has proven to be effective. However, CBT availability for adolescents with CFS is limited and requires special therapeutic skills not always readily available. An alternative to the face-to-face CBT is FITNET, a web-based therapeutic program designed specifically for adolescents diagnosed with CFS, and their parents. This new CBT approach appeals to the modern youth, who grow up with internet as their main source of information. A web-based program offers the opportunity to lower thresholds for the acceptance and realization of healthcare. This treatment can be activated at any chosen time. The communication between patient and therapist can elapse asynchronously. If effective, this web-based program would greatly increase the therapeutic accessibility.

**Methods/Design:**

A randomized clinical trial is currently conducted. One-hundred-forty adolescents aged 12-18 years diagnosed with CFS will be recruited and randomized to one of two groups: FITNET or usual care. After 6 months, the usual care group will have access to the FITNET program. Outcomes will be assessed at baseline, post intervention, and at 6 months follow-up. Primary outcome measures are school presence, fatigue severity, and physical functioning.

**Discussion:**

The FITNET study is the first randomized clinical trial which evaluates the effect of web-based CBT versus usual care in adolescents with CFS. The intervention is based on a theoretical existing model of CBT for patients with CFS. The results of this study will provide information about the possibility and efficacy of web-based CBT for adolescents with CFS and will reveal predictors of efficacy.

**Trial registration:**

ISRCTN: ISRCTN59878666 and ClinicalTrials.gov: NCT00893438

## Background

Chronic fatigue syndrome (CFS, also known as myalgic encephalomyelitis/encephalopathy or ME) is a disabling disorder, characterized by persistent or relapsing severe fatigue that is not the result of an organic disease or ongoing exertion and is not alleviated by rest. Symptoms last for at least 6 months and are accompanied by other symptoms like muscle pain and concentration problems[1].

The cause of CFS is still unknown. CFS is explained in terms of a central neurobiological disturbance with triggering, sustaining and perpetuating factors both on a biological and psychosocial level[2]. Cognitions concerning these sustaining and perpetuating factors are subject of the cognitive behavioural therapy (CBT) regimen for adolescents. CBT is the only evidence-based treatment with a recovery rate of 70% directly after treatment[3]. An uncontrolled study of the efficacy of family cognitive behaviour therapy for adolescents with CFS, revealed even better results with a recovery rate of 83%, with a maintained gain at 6 months follow-up[4]. However, the availability of this treatment in the Netherlands is limited and it requires special therapeutic skills that are not always readily available.

Furthermore, not all cases of CFS are adequately recognised, neither by the patient nor by their doctor. A large population study in the Netherlands revealed a prevalence rate of 16.4% of severe fatigue lasting more than one month in adolescent girls[5]. The proportion of undiagnosed cases of CFS among these girls is unclear. The lack of treatment possibilities within reach may enhance the reticence in diagnosing CFS.

Although untreated CFS in adolescents has a better prognosis than in adults, the risk of disruption of development and education asks for a prompt recognition of the syndrome and an effective treatment method[3,6]. The longest follow-up study, covering a 13 year follow-up period, showed that the majority of adolescents have mild to moderate persisting symptoms with a considerable period of school absence[6].

The lack of skilled cognitive behavioural therapists to treat adolescents with CFS made us decide to develop a web-based application combing CBT by a skilled therapist with a regular internet contact. In a recently published non-inferiority randomised controlled trial (RCT) by the Expert Centre for Chronic Fatigue (ECCF) in Nijmegen, a minimal intervention based on CBT for CFS, consisting of a self help booklet (workbook) supported by email contact with a cognitive behavioural therapist, appeared to be effective[7]. This Self Help Program resembles a web-based CBT in certain aspects, such as the absence of face-to-face contact between therapist and patient. However, in the minimal intervention study the contact between therapist and patient was minimal, whereas in the web-based CBT there will be frequent email contact between therapist and patient.

Web-based CBT for illnesses other than CFS has been found effective, but most research has been conducted with adults[8,9]. For adolescents, web-based CBT has been developed for disorders such as obesity [10], depression [11], anxiety [12], headache [13] and smoking cessation [14]. The efficacy of these web-based CBT programs in general is comparable with the face-to-face treatments[8,9,13,15].

FITNET (web-based treatment for Fatigue In Teenagers) is a Dutch web-based program developed in close collaboration between the Wilhelmina Children's Hospital (University Medical Centre Utrecht, UMCU) and the Expert Centre for Chronic Fatigue (Radboud University Nijmegen Medical Centre, ECCF). FITNET is a highly structured program for adolescents with CFS and consists of two parts: a psycho-educational part for both adolescents and parents and a CBT part based on the protocol of the ECCF[16]. The efficacy of this protocol in a face-to-face setting for adolescents has been demonstrated in a randomized controlled trial[3].

The primary aim of this study is to determine the efficacy of FITNET for adolescents with CFS in the Netherlands. The second goal of this study is to explore predictors of outcome.

## Methods/Design

### Study design

**Figure 1:**
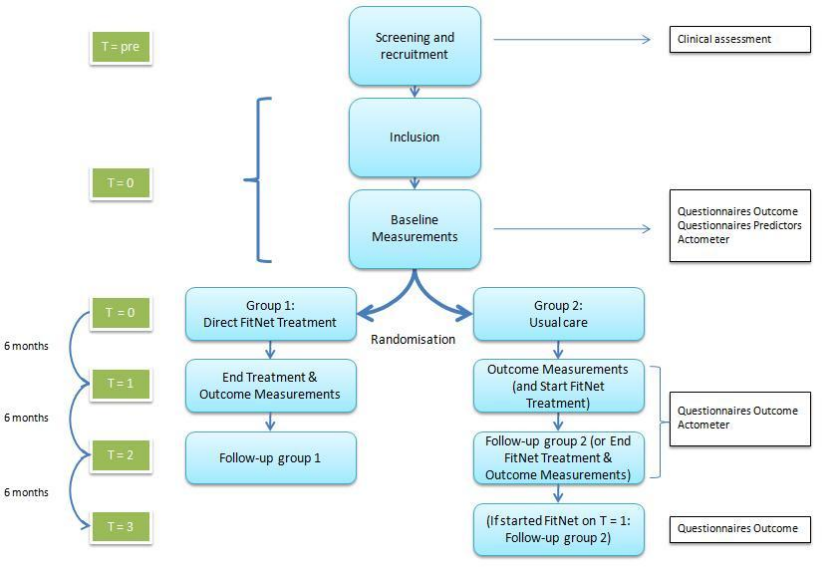
Flowchart of trial design.

A single-blinded randomized clinical trial (RCT) with 6 months follow-up will be conducted to evaluate the efficacy of FITNET compared to usual care for adolescents with CFS. Efficacy of FITNET compared to usual care will be determined after 6 months, the maximum duration of the treatment. Patients assigned to FITNET have to agree to not have any further medical examinations or other treatments for fatigue whilst in therapy[17]. The adolescents who have been assigned to the usual care will get the opportunity to attend the program after these 6 months. The total follow-up time is 12 months after the start of the web-based program. (*see Figure *1).

CFS will be diagnosed after a uniform diagnostic work-up by a paediatrician specialised in CFS (EP), according to CDC-criteria[1]. Once the diagnosis is established, study eligibility will be assessed by completing self-reported questionnaires on fatigue, physical complaints, physical functioning, depression and anxiety. Eligible patients will be asked to participate in this RCT. If the patient is willing to participate, the primary investigator (SN) will check the inclusion and exclusion criteria (*table *1). When a patient meets all criteria, oral and written consent will be obtained from both patient and at least one parent, according to the declaration of Helsinki. The patients with a possible psychiatric comorbidity and their parents will be examined by an experienced child psychologist before randomisation. Patients with a primary psychiatric diagnosis, as assessed by this psychologist, will be excluded from the trial. If the adolescent or the parent(s) decide not to participate in the study, the reason will be asked and specified, although they are not obligated to reveal such reasons. During the intervention we expect some adolescents to drop-out. The reason to stop will be asked and specified.

**Table 1:** Inclusion and exclusion criteria

Inclusion criteria
(1) The participant has given written informed consent

(2) CFS diagnosis according to the CDC criteria [1]

(3) Adolescent between 12-18 years old at inclusion

(4) Fatigue severity subscale (CIS-20) score ≥40 (healthy population's mean plus two SD) [19]

(5) Physical functioning (Child Health Questionnaire) score <85 (healthy population's mean minus two SD) and/or school participation ≤85% (healthy population's mean minus two SD) in last two weeks [20]

**Exclusion criteria**

(1) Inadequate control of Dutch language by child or parent

(2) No availability of computer hardware and internet connection

(3) Suicide risk as assessed on the Children's Depression Inventory (CDI) [21]

(4) Cognitive retardation (when indicated an IQ-test will be conducted; IQ <85 will be excluded)

(5) Score greater than or equal to 44 (healthy population's mean plus two SD) on the State-Trait Anxiety Inventory for Children (STAIC) [23]

(6) Score greater than or equal to 16 (healthy population's mean minus two SD) on the Children's Depression Inventory (CDI) [21]

### Study population

It is intended to include 140 adolescents aged 12-18 years old, referred to the University Medical Centre Utrecht (UMCU) and diagnosed with CFS using the CDC-criteria[1].

The patients will be recruited from second-line medical care by means of the National Surveillance Centre for Children (NSCK), which is part of the International Network of Pediatric Surveillance Units, INoPSU[18]. The NSCK survey guarantees a high percentage of reporting (83-92%) of the diagnosis under consideration. Recruitment from primary care will be realised by announcement of this study to all General Practitioners in the Netherlands (with help of the NIVEL, the Dutch Collaborating Centre of the WHO). In addition, on the website of the 'CVS-ME Stichting' (a Dutch CFS Foundation) a direct call to participate in this study will be published.

### Ethical approval

This study has been reviewed and approved by the Medical Ethical Committee of the University Medical Centre Utrecht (reference 07/196-K) and the Medical Ethical Committee of the Radboud University Nijmegen Medical Centre (reference AMO nr. 07/105). Patients and their parents receive verbal and written information about the study and informed consent will be obtained before randomisation.

### Randomization and blinding

Concealed randomisation will be performed by the data-management section of the Julius Centre for Health Sciences and Primary Care Utrecht. This randomization is computer-generated by creating 'blocks' with a size of 6, ensuring that the same number of participants will be allocated to each group. The investigators who are responsible for the inclusion will be blinded. Primary outcome parameters will be assessed by computer.

### Interventions

#### Usual care

The patients in the control group will receive the usual care available in the region where the patient lives. The available usual care for adolescents with CFS in the Netherlands includes: individual/group based rehabilitation programs, psychological support including CBT face-to-face, graded exercise therapy by a physiotherapist, etc. All care received will be monitored during the study.

#### Web-based Cognitive Behavioural Therapy (FITNET)

FITNET is a web-based cognitive-behavioural treatment accessible to patients and both parents, based on the existing face-to-face CBT protocol for adolescents developed by the ECCF [3,17]. Trained cognitive behavioural psychotherapists from the ECCF will support the patients by e-consults. There will be no face-to-face contact between the therapists and patients at all.

The web portal is designed by the UMCU in collaboration with the ECCF, and has been developed in cooperation with adolescents with CFS who critically appraised text, lay-out and structure. Two authors of children books (Mr. and Mrs. van Hagen) revised the textual content on readability for adolescents. All therapists receive a special half a day training in the application of written language for this target group.

The programme consists of a psycho-educational and a CBT section. The psycho-educational section will be readily available, after receiving log in codes. The CBT section consists of 21 interactive modules (*see appendix 1*), accessible upon activation by the therapist. The web-based therapy comprises about 20 internet-sessions over 6 months. Meanwhile, the parents will follow a parallel program. All users will have a unique username and password, ensuring private communication with the therapist. Both parents and adolescents will have a regular e-consult (email contact) with the therapist, wherein results so far are discussed and new assignments can be given. The therapist will reply on a fixed weekday. The patient will have the ability to send an emergency email, on which a prompt reply will be made. For emergency situations, telephone contact details are available for the patient.

The FITNET treatment programme is implemented in a portal with a lay-out especially designed for adolescents, combining different applications such as: e-consult, personal diaries, questionnaires, psycho-education, all treatment steps and the possibility to review previous communications and assignments. Patient data and emails will be encrypted and securely stored on the UMCU mainframes to guarantee privacy and confidentiality. The treatment protocol is described in more detail in the appendix.

### Compliance and attrition

Therapy compliance will be assessed by recording the number of treatment-modules (CBT) that have been completed. When applicable, participants will be asked for their reasons for poor compliance. In the case of therapy drop-out, patients will be asked for the reason of non compliance and stimulated to continue participation in the assessments until the last follow-up.

### Outcomes

Outcome measures and predictors of outcome are listed in table 2.

**Table 2:** Outcome measures, predictors of outcome and instrumentation

	Instruments	T0	T1	T2	T3
**Primary outcome parameters**					

Fatigue severity	Checklist Individual Strength (CIS subscale fatigue) [19]	X	X	X	X

Physical Functioning	Child Health Questionnaire (CHQ-CF87 subscale physical functioning) [20]	X	X	X	X

School presence	Last two weeks school presence expressed in attended hours/obliged hours * 100%[3]	X	X	X	X

**Secondary outcome parameters**					

Self rated improvement	short questionnaire consisting of 3 items [3]	X	X	X	X

**Possible patient's predictors of treatment outcome**					

Depression score	Child Depression Inventory (CDI) [21,22]	X			

Anxiety score	Spielberger State-Trait Anxiety Inventory for Children, STAIC [23,24]	X			

Somatisation score	Children's Somatisation Inventory (CSI) [25,26]	X			

Physical activity	Actometer and Self-observation list daily functioning [27,28]	X	X	X	

Self-efficacy	Self Efficacy Scale-28[2]	X			

Perceptions of parental rearing behaviours	EMBU-A [29]	X			

**Possible parental predictors of treatment outcome**					

Fatigue severity	Checklist Individual Strength (CIS subscale fatigue) [19]	X			

Psychological distress	Symptom Checklist (SCL-90) [30,31]	X			

Focussing on bodily symptoms	subscale private body consciousness of the Body Consciousness Scale [32,33]	X			

Perceptions of rearing behaviours	EMBU-P [34]	X			

Causal attributions	CAL [19]	X			

The primary outcome measures are: (1) Fatigue as measured by the subscale *fatigue severity *(8 items, 7-points Likert Scale) of the Checklist Individual Strength-20 [19] with a severity range from 8-56. The questionnaire has good reliability and discriminative validity. (2) Physical functioning as measured by the subscale *physical functioning *(9 items) of the validated Dutch translation of the Child Health Questionnaire (CHQ-CF87) (0-100%) [20], and (3) School presence (expressed in attended hours/obliged hours * 100%) as assessed in prior intervention studies in adolescents with CFS[3]. Secondary outcome measure is self-rated improvement, measured using a short questionnaire consisting of 4 items as assessed in prior intervention studies in adolescents with CFS [3]: patients indicate whether they have completely recovered, feel much better, have the same complaints of have become worse compared with the previous measurement.

Outcome measures will be obtained at the UMCU at the start of the study period (T0), after the intervention 6 months later (T1) and after 6 months follow-up (T2). For those adolescents primarily allocated to usual care who will start with FITNET at T1 - in case of no recovery - there will be an extra follow-up moment after 12 months (T3), *see figure *1.

At the first measurements (T0) demographic data and the following predictors of response to treatment and measures of processes of change will be obtained by the primary investigator (SN) both for adolescent and parent(s) *(see table *2*)*. Possible predictors of response to treatment in adolescents are: (a) Depression score (validated Dutch translation of the Child Depression Inventory, CDI) [21,22], (b) Anxiety (Dutch translation of the Spielberger State-Trait Anxiety Inventory for Children, STAIC) [23,24], (c) Number and severity of other somatic symptoms measured by a validated Dutch translation of the Children's Somatisation Inventory (CSI) [25,26], (d) Physical performance as measured with the actometer. This is a motion-sensing device worn at the ankle that registers and quantifies physical activity. The actometer is worn day and night during a period of twelve consecutive days [27]. During the same days patients rate fatigue, pain and activity levels on a prescheduled diary four times daily on a scale of 0 (not at all) to 4 (very much). Daily registration of hours being at school is registered as well. The psychometric qualities are good [28], (e) Self-efficacy (Self Efficacy Scale-28) [2], (f) Perceptions of parental rearing behaviours (the adolescent version of the Egna Minnen Beträffende Uppfostran, EMBU-A) [29].

Possible parental predictors of response to treatment are: (a) Parental fatigue (Checklist Individual Strength-20) [19], (b) Parental psychological distress measured by the Symptom Checklist (SCL-90) [30,31], (c) Parental focussing on bodily symptoms by the subscale private body consciousness of the Body Consciousness Scale [32,33], (d) EMBU-P, the parental version of the Egna Minnen Beträffende Uppfostran [34], (e) parental causal attributions to the origin of CFS (Causal Attribution List, CAL) [19,28].

Finally, the patients and parent(s) treated with FITNET (including the drop-outs) will be interviewed (responsive evaluation) about their experiences with the FITNET intervention. The interviews are semi-structured, with open questions guided by a topic list (10 point scale, questionnaire especially developed for this study). Different aspects of this web-based programme will be evaluated: text, lay-out, feedback by the therapist, scheduling of modules, technical experience, etc.

### Adverse events

The delivery of CBT to adolescents and adults is considered safe.

All adverse events reported spontaneously by the participants or observed by the therapists will be recorded. All adverse events will be followed until they have aborted, or until a stable situation has been reached.

### Statistical Analysis

One-hundred-forty newly diagnosed CFS patients will be included in two years. The data will be analysed on an intention to treat basis. Prognostically important baseline characteristics will be tabulated by treatment modality in order to evaluate success of randomisation. The main efficacy analysis will pertain to the data obtained after a 6 months FITNET or usual care condition. Differences between treatment groups concerning the primary outcome measures will be expressed as relative risks and 95% confidence intervals or as central estimators and variance measures where appropriate. In case of baseline differences in prognosis between groups, treatment effects in primary outcome will additionally be adjusted for these differences using logistic regression for binary outcome data and linear regression for continuous outcome measures. At 12 months follow-up after randomization, a repeated measurement of the primary outcome measures will be undertaken as well as an evaluation of participants' experiences with the FITNET intervention. The latter measurements will have a descriptive nature.

Concerning the predictors of outcome, the study will be merely exploratory because statistical power is not calculated for subgroup analyses. To that end, linear regression analysis will be used with primary continuous outcome measures as dependent variables and interaction terms of putative response predictors and treatment modality as independent variables. Separate analyses will be run for predictors in adolescents and predictors in parents respectively.

### Power

We estimate that under care as usual the estimated average percentages of school absence, our primary endpoint, at the end of follow-up, will be 40%. Given the efficacy of face to face CBT in adolescence [3] we consider a reduction of school absence of 15% in the e-intervention group to be realistic and highly relevant. In order to statistically detect such a reduction, given a 2 sided-alpha of 0.05 and with 90% power, we will need to allocate 60 patients to each group. In order to accommodate a small anticipated percentage of non-participation we will include 140 diagnosed patients.

## Discussion

To the best of our knowledge, the FITNET study will be the first randomized clinical trial which evaluates the effect of web-based treatment versus usual care in adolescents with CFS.

This study has several strengths. Firstly, the FITNET treatment is based on a theoretical model of a proven effective face-to-face CBT for adolescents [3] and will be compared with usual care in a randomized design. This will enable to determine the additional value of FITNET in the current therapeutical spectrum. Secondly, FITNET involves parents in the treatment of adolescent CFS. Earlier studies from Chalder et al showed the importance of family-focused CBT to achieve treatment success in adolescents [4,35]. Furthermore, the advantage of making treatment available at any time by internet is considerable. Online treatment can reduce face-to-face treatment barriers (i.e., inconvenience of scheduling appointments, missing school/work, travelling to and from a clinician's office) [36,37], increase adherence [38] and reduce treatment time and costs[37]. We hope that a web-based treatment will increase the therapeutic accessibility. A limitation of the study is that the design is not appropriate to compare the efficacy of web-based CBT with face-to-face CBT. That would have asked for a non-inferiority design with comparison of the two interventions.

In conclusion, the FITNET study will provide greater insight on evidence-based treatment options for adolescents with CFS. If web-base CBT is more effective than usual care, this web-based program would greatly improve the prognosis of the adolescents with CBT.

## Abbreviations

CBT: Cognitive Behavioural Therapy; CDC: Centre for Disease Control and Prevention; CFS: Chronic Fatigue Syndrome; CIS-20: Checklist Individual Strength; CHQ-CF87: Health Questionnaire-Child Form; ECCF: Expert Centre for Chronic Fatigue; RCT: Randomized Controlled Trial; UMCU: University Medical Centre Utrecht.

## Competing interests

The authors declare that they have no competing interests.

## Authors' contributions

SN is primary investigator and responsible for data collection and analysis and for drafting the manuscript. EP, GB, CU, and JK designed and supervised the study. EP obtained funding for the study. All authors have read and approved the final manuscript.

## Appendix 1: Overview of FITNET web-based CBT treatment for adolescents with CFS

### *Psycho-educational part*

What is CFS?

CFS in the Netherlands

CFS in my family

Causes of CFS

CFS, anxiety, depression and other illnesses

How is the diagnosis confirmed?

What is the treatment for CFS?

Talking about CFS/How do I explain what CFS is?

Future forecast

### Cognitive Behavioural Treatment Modules

1. To introduce myself

2. How does this treatment work?

3. Assessing my present possibilities

4. My parents

5. My goals

6. My sleep routine

7. My thoughts

8. Changing my attention to fatigue

9. Step up my physical activities (*passive patients*)

10. Balance between activity and rest (*relative active patients*)

11. Step up my physical activities (*relative active patients*)

12. Recognizable problems with the treatment

13. Step up my mental activities

14. My schedule for school

15. My social activities

16. To reach goals

17. My schedule for work

18. To have a night out

19. Do I still see myself as a patient with CFS?

20. My evaluation

21. Follow-up

FITNET is derived from the protocol for CBT that was developed on the basis of a model of perpetuating factors of CFS[2]. It has been tested in several studies [3,17,39] and is aimed at changing fatigue related cognitions and a gradual increase of activities.

Typically, treatment involves (a) formulation of treatment goals, (b) establishing a sleep routine, (c) encouraging the participant to achieve a balance between activity and rest, (d) gradually increasing activities including home, social and school life, (e) addressing cognitions about fatigue, (f) gaining independence from surroundings and parents, (g) reducing the focus on fatigue, and (h) paying attention to relapse prevention.

Addressing physical activity patterns is important in CBT for adolescent CFS. The CBT modules are adapted for two levels of physical activity: relative active and passive, based on the existing protocols[3,16,17]. An actometer, a motion sensing device that can quantify physical activity, will be used to assess the activity pattern [27,32]. Adolescents with a *relatively high physical activity pattern *alternate between periods of activity and periods of rest[16]. For these patients the therapy focuses on learning to recognise and accept their current state of fatigue and impairment. Then they learn to distribute their activities more evenly. After this, the patient will start to build up activity levels. In contrast, patients with a *low physical activity pattern *spend most time lying down and go out infrequently. Most do not attend school at all. For them, the treatment program starts with a systematic build up of activity as soon as possible, while addressing and challenging their beliefs that activity would aggravate symptoms.

Modules 1, 2 and 4 introduce CBT and explain the role of the therapists, and the context of the family. A rationale based on a multi-factorial model of CFS that distinguished predisposing, precipitating and maintaining factors is presented. Parents will be actively involved in supporting their child and parents' beliefs and behaviours regarding the condition of their child will be explored and addressed. The aims of therapy take into account the specific developmental tasks of adolescents. In children younger than 15 years, parents often act as a coach; for older participants, parents have to step back and encourage their child to take responsibility for the treatment.

Modules 3 and 5 are focused on treatment goals. Return to full time education is always a goal of treatment, and a plan for returning to school will be discussed early with everyone involved.

Modules 6 to 19 focus on cognitive behavioural strategies and include instructions and exercises on how to identify, challenge and change cognitive processes that contribute to CFS. There are two treatment protocols, depending on the pattern of physical activity of the patient[16].

Module 20 evaluates treatment success.

Module 21 is a relapse preventing module focusing on maintaining gains and staying healthy.

### Diaries

Sleep diary, Helpful Thoughts, My goals, Activity schedule, School schedule

## Pre-publication history

The pre-publication history for this paper can be accessed here:

http://www.biomedcentral.com/1471-2377/11/23/prepub

## References

[B1] Fukuda K Straus SE Hickie I Sharpe MC Dobbins JG Komaroff A The chronic fatigue syndrome: a comprehensive approach to its definition and study. International Chronic Fatigue Syndrome Study Group Ann Intern Med 1994 121 12 953 959 7978722 10.7326/0003-4819-121-12-199412150-00009

[B2] Vercoulen JH Swanink CM Galama JM Fennis JF Jongen PJ Hommes OR van der Meer JW Bleijenberg G The persistence of fatigue in chronic fatigue syndrome and multiple sclerosis: development of a model J Psychosom Res 1998 45 6 507 517 10.1016/S0022-3999(98)00023-3 9859853

[B3] Stulemeijer M de Jong LW Fiselier TJ Hoogveld SW Bleijenberg G Cognitive behaviour therapy for adolescents with chronic fatigue syndrome: randomised controlled trial BMJ 2005 330 7481 14 10.1136/bmj.38301.587106.63 15585538 PMC539840

[B4] Chalder T Tong J Deary V Family cognitive behaviour therapy for chronic fatigue syndrome: an uncontrolled study Arch Dis Child 2002 86 2 95 97 10.1136/adc.86.2.95 11827901 PMC1761081

[B5] ter Wolbeek M van Doornen LJ Kavelaars A Heijnen CJ Severe fatigue in adolescents: a common phenomenon? Pediatrics 2006 117 6 e1078 e1086 10.1542/peds.2005-2575 16740810

[B6] Bell DS Jordan K Robinson M Thirteen-year follow-up of children and adolescents with chronic fatigue syndrome Pediatrics 2001 107 5 994 998 10.1542/peds.107.5.994 11331676

[B7] Tummers M Knoop H Bleijenberg G Effectiveness of stepped care for chronic fatigue syndrome: a randomized noninferiority trial J Consult Clin Psychol 2010 78 5 724 731 10.1037/a0020052 20873907

[B8] Emmelkamp PM Technological innovations in clinical assessment and psychotherapy Psychother Psychosom 2005 74 6 336 343 10.1159/000087780 16244509

[B9] Cuijpers P van SA Andersson G Internet-administered cognitive behavior therapy for health problems: a systematic review J Behav Med 2008 31 2 169 177 10.1007/s10865-007-9144-1 18165893 PMC2346512

[B10] Williamson DA Martin PD White MA Newton R Walden H York-Crowe E Alfonso A Gordon S Ryan D Efficacy of an internet-based behavioral weight loss program for overweight adolescent African-American girls Eat Weight Disord 2005 10 3 193 203 16277142 10.1007/BF03327547

[B11] O'Kearney R Gibson M Christensen H Griffiths KM Effects of a cognitive-behavioural internet program on depression, vulnerability to depression and stigma in adolescent males: a school-based controlled trial Cogn Behav Ther 2006 35 1 43 54 16500776 10.1080/16506070500303456

[B12] Spence SH Holmes JM March S Lipp OV The feasibility and outcome of clinic plus internet delivery of cognitive-behavior therapy for childhood anxiety J Consult Clin Psychol 2006 74 3 614 621 10.1037/0022-006X.74.3.614 16822117

[B13] Trautmann E Kroner-Herwig B A randomized controlled trial of Internet-based self-help training for recurrent headache in childhood and adolescence Behav Res Ther 2009 19782343 10.1016/j.brat.2009.09.004

[B14] Patten CA Croghan IT Meis TM Decker PA Pingree S Colligan RC Dornelas EA Offord KP Boberg EW Baumberger RK Hurt RD Gustafson DH Randomized clinical trial of an Internet-based versus brief office intervention for adolescent smoking cessation Patient Educ Couns 2006 64 1-3 249 258 10.1016/j.pec.2006.03.001 16616449

[B15] Cuijpers P Marks IM van Straten A Cavanagh K Gega L Andersson G Computer-aided psychotherapy for anxiety disorders: a meta-analytic review Cogn Behav Ther 2009 38 2 66 82 10.1080/16506070802694776 20183688

[B16] Bleijenberg G Prins J Bazelmans E Jason LA, Fennel PA, Taylor RR Cognitive behavioral therapies Handbook of Chronic Fatigue Syndrome 2003 Wiley & Sons, Inc.Hoboken 493 526

[B17] Prins JB Bleijenberg G Bazelmans E Elving LD de Boo TM Severens JL van der Wilt GJ Spinhoven P van der Meer JW Cognitive behaviour therapy for chronic fatigue syndrome: a multicentre randomised controlled trial Lancet 2001 357 9259 841 847 10.1016/S0140-6736(00)04198-2 11265953

[B18] Hira Sing RA Rodrigues PR [The Dutch Pediatric Surveillance System; a quality focused instrument for prevention and research] Ned Tijdschr Geneeskd 2002 146 50 2409 2414 12518518

[B19] Vercoulen JH Swanink CM Fennis JF Galama JM van der Meer JW Bleijenberg G Dimensional assessment of chronic fatigue syndrome J Psychosom Res 1994 38 5 383 392 10.1016/0022-3999(94)90099-X 7965927

[B20] Raat H Landgraf JM Bonsel GJ Gemke RJ Essink-Bot ML Reliability and validity of the child health questionnaire-child form (CHQ-CF87) in a Dutch adolescent population Qual Life Res 2002 11 6 575 581 10.1023/A:1016393311799 12206578

[B21] Kovacs M The Children's Depression, Inventory (CDI) Psychopharmacol Bull 1985 21 4 995 998 4089116

[B22] Timbremont B Braet C Psychometrische evaluatie van de Nederlandstalige Children's Depression Inventory Gedragstherapie 2001 34 3 229 242

[B23] Papay JP Spielberger CD Assessment of anxiety and achievement in kindergarten and first- and second-grade children J Abnorm Child Psychol 1986 14 2 279 286 10.1007/BF00915446 3722623

[B24] Houtman IL Bakker FC The anxiety thermometer: a validation study J Pers Assess 1989 53 3 575 582 10.1207/s15327752jpa5303_14 2778618

[B25] Meesters C Muris P Ghys A Reumerman T Rooijmans M The Children's Somatization Inventory: further evidence for its reliability and validity in a pediatric and a community sample of Dutch children and adolescents J Pediatr Psychol 2003 28 6 413 422 10.1093/jpepsy/jsg031 12904453

[B26] van de Putte EM Engelbert RH Kuis W Sinnema G Kimpen JL Uiterwaal CS Chronic fatigue syndrome and health control in adolescents and parents Arch Dis Child 2005 90 10 1020 1024 10.1136/adc.2005.074583 16049059 PMC1720106

[B27] van der Werf SP Prins JB Vercoulen JH van der Meer JW Bleijenberg G Identifying physical activity patterns in chronic fatigue syndrome using actigraphic assessment J Psychosom Res 2000 49 5 373 379 10.1016/S0022-3999(00)00197-5 11164063

[B28] Vercoulen JH Hommes OR Swanink CM Jongen PJ Fennis JF Galama JM van der Meer JW Bleijenberg G The measurement of fatigue in patients with multiple sclerosis. A multidimensional comparison with patients with chronic fatigue syndrome and healthy subjects Arch Neurol 1996 53 7 642 649 8929171 10.1001/archneur.1996.00550070080014

[B29] Gerlsma C Arrindell WA Veen van der N Emmelkamp PM A parental rearing style questionnaire for use with adolescents: Psychometric evaluation of the EMBU-A Personality and Individual Differences 1991 12 1245 1253 10.1016/0191-8869(91)90196-I

[B30] van de Putte EM van Doornen LJ Engelbert RH Kuis W Kimpen JL Uiterwaal CS Mirrored symptoms in mother and child with chronic fatigue syndrome Pediatrics 2006 117 6 2074 2079 10.1542/peds.2005-2307 16740850

[B31] Derogatis LR SCL-90-R administration, scoring & procedures. Manual I. For the R(evised) version and other instruments of the psychopathology rating scale series Clinical Psychometric Research 1977

[B32] Bazelmans E Bleijenberg G Voeten MJ van der Meer JW Folgering H Impact of a maximal exercise test on symptoms and activity in chronic fatigue syndrome 2 J Psychosom Res 2005 59 4 201 208 10.1016/j.jpsychores.2005.04.003 16223622

[B33] van der Werf SP de Vree B Der Meer JW Bleijenberg G The relations among body consciousness, somatic symptom report, and information processing speed in chronic fatigue syndrome Neuropsychiatry Neuropsychol Behav Neurol 2002 15 1 2 9 11877546

[B34] Arrindell WA Perris C Van der EJ Gaszner P Eisemann M Perris H Cross-national generalizability of dimensions of perceived parental rearing practices: Hungary and The Netherlands; a correction and repetition with healthy adolescents Psychol Rep 1989 65 3 Pt 2 1079 1088 2623099 10.2466/pr0.1989.65.3f.1079

[B35] Chalder T Deary V Husain K Walwyn R Family-focused cognitive behaviour therapy versus psycho-education for chronic fatigue syndrome in 11- to 18-year-olds: a randomized controlled treatment trial Psychol Med 2010 40 8 1269 1279 10.1017/S003329170999153X 19891804

[B36] Ritterband LM Thorndike FP Cox DJ Kovatchev BP Gonder-Frederick LA A behavior change model for internet interventions Ann Behav Med 2009 38 1 18 27 10.1007/s12160-009-9133-4 19802647 PMC2878721

[B37] Tate DF Finkelstein EA Khavjou O Gustafson A Cost effectiveness of internet interventions: review and recommendations Ann Behav Med 2009 38 1 40 45 10.1007/s12160-009-9131-6 19834778 PMC2772952

[B38] Celio AA Winzelberg AJ Wilfley DE Eppstein-Herald D Springer EA Dev P Taylor CB Reducing risk factors for eating disorders: comparison of an Internet- and a classroom-delivered psychoeducational program J Consult Clin Psychol 2000 68 4 650 657 10.1037/0022-006X.68.4.650 10965640

[B39] Knoop H Bleijenberg G Gielissen MF van der Meer JW White PD Is a full recovery possible after cognitive behavioural therapy for chronic fatigue syndrome? Psychother Psychosom 2007 76 3 171 176 10.1159/000099844 17426416

